# The Impact of COVID on Cat Guardians: Veterinary Issues

**DOI:** 10.3390/ani11030603

**Published:** 2021-02-25

**Authors:** Lori R. Kogan, Phyllis Erdman, Jennifer Currin-McCulloch, Cori Bussolari, Wendy Packman

**Affiliations:** 1 Clinical Sciences, Colorado State University, Fort Collins, CO 80523, USA; 2 College of Education, Washington State University, Pullman, WA 99163, USA; perdman@wsu.edu; 3 School of Social Work, Colorado State University, Fort Collins, CO 80523, USA; jen.currin-mccolloch@colostate.edu; 4 Counseling Psychology, University of San Francisco, San Francisco, CA 94117, USA; bussolari@usfca.edu; 5 Department of Psychology, Palo Alto University, Palo Alto, CA 94304, USA; wpackman@paloaltou.edu

**Keywords:** COVID, feline, cat, veterinary, protocol, veterinary care

## Abstract

**Simple Summary:**

COVID-19 has impacted veterinary medicine and cat guardians in numerous ways. The purpose of this study was to better understand cat guardians’ fears and concerns pertaining to veterinary care and the ability to obtain pet care products and food during the initial months of the COVID pandemic. We distributed an anonymous online survey to cat guardians that included questions pertaining to guardians’ relationship with their cat and their veterinary related concerns and experiences as a result of the pandemic. The results, from 956 participants revealed that the increased amount of time guardians spent with their cat had a positive impact on their bond. Participants’ primary veterinary related concerns centered around the availability of their veterinarian for both emergency and non-emergency care. Other concerns they shared included fears about the ability to afford emergency veterinary care and obtain cat food and supplies. Awareness of these concerns can help veterinarians better meet the needs of cat guardians by directly communicating their continued availability and presence in the face of a pandemic as well as other challenging times.

**Abstract:**

The onset of COVID has impacted the field of veterinary medicine and the lives of cat guardians in numerous ways, yet the subject remains largely unexplored. The purpose of this study was to better understand cat guardians’ fears and concerns pertaining to veterinary care and obtainment of pet care products and food during the initial lock down phase of the COVID pandemic to better address these concerns now and in the future. To this end, an anonymous online survey was distributed to cat guardians during the first two months of the pandemic. The survey included questions pertaining to guardians’ relationship with their cat and their veterinary related concerns and experiences as a result of the pandemic. Quantitative and qualitative data were collected from 956 participants. The results revealed that the increased amount of time guardians spent with their cat had a positive impact on their bond. Participants’ veterinary related concerns, particularly for participants between 18–29 years of age, centered around availability of their veterinarian for both emergency and non-emergency care. Other concerns included fears about the ability to afford emergency veterinary care and obtain cat food and supplies. Awareness of these concerns can help veterinarians better support cat guardians by directly communicating their continued availability and presence in the face of a pandemic as well as other challenging times.

## 1. Introduction

An estimated 67% of US households have at least one pet, and 38% of these homes include at least one cat, leading to approximately 94.2 million companion cats in the US [1]. These figures are comparable with Canada, where 37% of homes have a cat [2]. While still substantial, although slightly lower, 27% of Australian homes have a cat [3] and in the UK, 24% of adults have a companion cat [4]. Cats are also common in Brazil, with 17.7% of homes containing at least one cat [5]. Regardless of geographical location, the majority of cat guardians reported feeling that their cat is a family member and that the positives of having a cat outweighed any negative aspects, with key benefits including love, affection, companionship, and the ability to make their guardians happy [3,4,6]. Yet, with these benefits comes responsibility, not least of which is the provision of veterinary care [2,3].

Although dogs and cats are the most common type of companion animals [1] and share some similarities, there are many differences between the two species, including how they are perceived, how they interact with their guardians, and the characteristics of guardians themselves. Cats interact with people differently than dogs; cat-human interactions typically involve signals used in cat–cat encounters with some modifications. For example, meows and purring are often employed as attention-seeking vocalizations that are encouraged and rewarded by cat guardians [7]. Another form of interspecific communication between cat and guardian involves the eye. The slow blink, involving a series of half-blinks followed by an extended narrowing of the eye or a full eye closure, has been found to be a form of cat–owner communication [8]. So, while it is increasingly clear that cats communicate regularly with their guardians, perhaps due to their divergent domestication histories [9], these forms of communication can be quite subtle and not as easily read and interpreted as overt dog communication [7]. Another important distinction between dogs and cats is how they are typically acquired. As Dotson and Hyatt noted, people typically proactively acquire a dog, while many cat guardians report feeling that their cat chose them [10]. In addition, animal guardians appear to place a higher economic value on dogs compared to cats, evidenced by a willingness to pay more for veterinary care [11]. 

Beyond differences between interspecies communication and interaction, there appear to be personality differences between cat and dog guardians [12], with dog guardians seen as more extraverted, conscientious, and agreeable than cat guardians [13]. Furthermore, a recent study exploring differences between dog and cat guardians found that dog guardians reported higher self-esteem than cat guardians or people without companion animals, while cat guardians reported lower self-esteem than people without companion animals [14]. These studies suggest that there are many differences between the interspecific relationship of cat/human and dog/human, as well as the characteristics of dog and cat guardians. It is therefore hypothesized that cat guardians may respond differently than dog guardians to the veterinary related challenges associated with the COVID pandemic. 

The onset of COVID in March 2020 brought countless changes which immediately impacted the field of veterinary medicine. In many countries, including the UK, the US, Australia and Canada, and throughout Europe, veterinary hospital protocols were suddenly altered as attempts were made to continue offering veterinary care in as safe a way as possible. Common changes include limiting services to essential/emergencies, a shift to ‘curbside care’ in which clients wait outside while their pet is taken inside by veterinary professionals, and different forms of telemedicine [15,16,17,18]. 

Entities like the Center for Disease Control and Prevention (CDC) published the *Interim Infection Prevention and Control Guidance for Veterinary Clinics Treating Companion Animals* in April 2020, and subsequently has provided frequent updates (the latest update was in August 2020) [19]. The report is designed to provide guidance to veterinarians and their staff who are treating or advising on companion animal medical care during the pandemic, with the intent of “*facilitating preparedness and to ensure practices are in place in a companion animal veterinary clinical setting to help people and animals stay safe and healthy*”. Yet, even with these CDC suggestions regarding procedural strategies [20], studies found significant variation in how veterinary hospitals interpreted and implemented these suggested policies during the first several months of COVID [21]. Faced with an increased demand for services, as well as staffing shortages, most veterinary hospitals continued to provide care, but others that were not able to provide care referred cases back to the guardians’ primary care veterinarian or provided minimal treatment with additional monitoring by the guardian. Hospitals also reported dealing with issues of safety protocols, shortages of staff, and financial impact [22]. 

In addition, the AVMA, as well as similar entities in other countries also worked to educate veterinary professionals and pet guardians about the risk of pets infecting humans and visa versa [23,24,25]. Because, in addition to the uncertainty surrounding veterinary care services, the early months of COVID were also marked by uncertainty regarding the possibility of passing COVID from human to companion cats or from cats to their guardians. While these publications and subsequent studies focused on suggested and implemented operational strategies and helped to provide information on how to plan and prepare for possible next steps, none addressed pet guardians’ concerns.

One of the first studies pertaining to veterinary clients’ perspectives was conducted by Banfield Pet Hospital [26]. In this study, a sample of 1,000 dog and cat guardians responded to a range of questions regarding how their personal wellbeing and concerns about their pets’ health had been impacted by COVID. A third of respondents reported feeling more attuned to their dog or cat than before the pandemic began, and 37% said they were paying more attention to their pet’s personal care. They also found that 41% of people said they had contacted their veterinarian during the early months of COVID related quarantine and Banfield noted that their call line, a service providing guardians with general pet care advice and triage support, caseload had increased nearly 90% since before COVID [27]. 

Another recent study found that while having pets during the lockdown phase of COVID brought people many benefits, it also created some stressors, including the ability to acquire pet supplies and concerns related to accessing veterinary care. In this study, US pet guardians indicated concern accessing veterinary care when needed, as well as trepidation regarding new curbside protocols at veterinary clinics that do not allow pet guardians to accompany their pets during appointments [28]. 

These findings suggest that the early quarantine months of COVID impacted pet guardians in many critical areas, yet the subject remains largely unexplored. Understanding the impact of COVID on cat guardians can help veterinary professionals best address their clients’ concerns and needs during a pandemic or other form of crisis. Therefore, the purpose of this study was to better understand cat guardians’ fears and concerns pertaining to veterinary care and obtainment of pet care products and food during the lock down phase of the COVID pandemic to be better prepared to address these concerns now and in the future. 

## 2. Materials and Methods

An online, anonymous, cross-sectional survey (in Supplementary Materials) was developed using Qualtrics (Qualtrics, Inc., Provo, UT, USA). The survey was designed, reviewed, and tested by the co-investigators and their colleagues (veterinary and social science professionals) after seeking input from cat-owning representatives from the community. The survey was pilot tested for ambiguity and/or potentially missing or inappropriate response options, with revisions made based on the results of the pilot testing. The final survey (see Supplementary Materials) and study design were approved by the Colorado State University Institutional Review Board (IRB # 20-10003H). Survey respondents were recruited through social media outlets and human animal focused organizations (e.g., Facebook cat-focused groups, Human Animal Interaction Section of American Psychological Association) between 30 March 2020 to 1 May 2020.

In order to maximize our understanding of the impact of COVID, cat guardians over the age of 18 from any country were encouraged to participate with the recognition that countries’ veterinary services (within countries as well as between) might be impacted differently. Demographic data were collected (e.g., age group, gender, country of residence, current level of COVID restrictions, number of adults, children and cats living in the home). Respondents were asked to indicate their social support before COVID and at the time they completed the survey and questions about the psychological impact of their cats during COVID restrictions (analyses of these items not included in this paper). Additionally, they were asked about changes in how much time they spent with their cat (in general and actively engaged). Next, they were asked about their perception of any changes in the bond they have with their cat before COVID and currently with two bond rating questions that asked them to rate their bond with their cat on a scale from 1 (not at all bonded) to 10 (extremely bonded). For people who indicated they had more than one cat, they were instructed to answer the survey questions for their cat whose name starts with a letter closest to the beginning of the alphabet (“for example, choose to answer about Fluffy rather than Sylvester since "F" comes before "S" in the alphabet”).

The next section of the survey included questions related to veterinary care and non-veterinary cat related concerns using a 4-point Likert scale (no concern to great concern). Veterinary related questions included concern level about their ability to afford veterinary care (emergency and non-emergency both now and in the future) and concern about the availability of their veterinarian for emergency and non-emergency issues. Additional veterinary related questions asked about their veterinary care experiences during COVID restrictions.

The next set of questions pertained to cat supplies (affordability and access), caretaker concerns, and zoonotic risks. These questions included the ability to afford or acquire cat food/supplies now or in the future. Guardians were also asked if they had designated a caretaker in case they were unable to care for their cat in addition to concern level for the ‘ability to play with your cat if you get COVID’ and ‘ability to keep your cat because of changes due to COVID’. 

Potential changes in guardians’ perceptions of cat behaviors were assessed with two questions. The first question asked guardians to indicate, right before the COVID-19 outbreak, how frustrated they felt about their cats’ undesirable behaviors on a scale from 1 (minimal/no frustration) to 10 (extremely frustrated). They could also select ‘no undesirable behaviors’. This question was followed with a similar question asking guardians to rate their current frustration level with respect to their cat’s behaviors. 

Data were analyzed using SPSS (IBM, Armonk, NY, USA). Descriptive statistics were calculated to characterize current attitudes and patterns in cat care during COVID restriction times. Paired *t*-tests were used to assess changes in bond score and frustration level with behaviors from one month before COVID to ‘current’ time. Associations between demographic variables and concern related questions were investigated using Kruskal-Wallis nonparametric analysis of variance. Significance level (α) was set at *p* = 0.05 and all tests were two-tailed.

## 3. Results

The total number of responses to the survey was 956, with the largest number of respondents from the United States. At the time of this survey, most participants reported restrictions in their city at the level of ‘all non-essential stores/businesses closed and ordered/strongly recommended to stay at home’ (Table 1). 

When queried about the number of people and cats in the home, the most common response for number of adults was two (489, 51.2%), followed by one (339, 35.5%). The majority of participants did not have children under the age of 18 living at home (868, 90.8%), followed by one child (65, 6.8%). Most participants owned two cats (318, 33.3%) with many others reporting owning one cat (294, 30.8%) or three cats (134, 14.0%). 

The age range of participants (*n* = 938) was 18–86 (X = 53.88 (SD 3.94)). For analysis, ages were categorized into younger than 30 (69, 7.4%), 30–39 (109, 11.6%), 40–49 (144, 15.4%), 50–59 (253, 27.0%), and 60 and older (363, 38.7%). The majority of respondents were female (975, 91.9%), with 74 (7.8%) males and 3 (0.3%) identifying as non-binary (4 did not answer). 

### 3.1. Bond and Activities with Cat

Participants were asked to indicate their bond level with their cat both one month before COVID and ‘currently’ on a scale of 1 (not bonded at all) to 10 (extremely bonded). The mean score for one month before was 8.98 (SD 1.55) and ‘currently’ was 9.28 (SD 1.27) (paired *t*-test = 10.31, *p* < 0.001). Cat guardians were asked to reflect on their frustration level with their cat’s undesirable behaviors one month prior to COVID and ‘currently’ on a scale of 1 (minimal/no frustration) to 10 (extremely frustrated) (or they could select no undesirable behaviors). The mean score for one month prior was 2.36 (SD 3.02) and ‘currently’ was 2.44 (SD 3.03). This difference was not significant (t = 0.12, *p* < 0.906). 

When asked whether COVID restrictions had changed the amount of time they spent with their cat, the majority reported spending more time with their cat (616, 64.4%), while 327 (34.2%) reported the time had not changed and 13 (1.4%) reported less time. For those who reported spending more time with their cat, they were asked if this increased time strengthened, strained, or both strengthened and strained the bond. Over half indicated it strengthened the bond (374, 61.6%), 157 (25.9%) reported no change, 67 (11.0%) reported strengthened and strained, and 9 (1.5%) reported strained (9 did not respond). 

### 3.2. Veterinary-Related Iissues

Participants were asked to indicate their concern level with several veterinary issues (*n* = 935). These items included the ability to afford emergency and non-emergency care now and in the future, concerns that their veterinarian would not be available for emergencies or non-emergencies, and concern about needing to leave the house if their cat required veterinary care. The issues of most concern were whether their veterinarian would be available if they needed them for emergencies and non-emergencies (Table 2). Kruskal-Wallis nonparametric analysis of variance was used to assess for age differences in stated concern level for these veterinary related issues (excluding those who responded NA). Participants under 30 years of age, compared to older guardians, were significantly more concerned that their veterinarian will not be available if they needed him/her for emergencies (*n* = 871) (H = 11.50, *p* = 0.022) and less concerned about the ability to afford non-emergency care in the future (*n* = 862) (H = 17.41, *p* = 0.002) No other concerns were significantly different based on age.

Participants were also asked to indicate their concern level with several animal, non-veterinary-related issues (*n* = 935). These included the ability to afford or acquire cat food/supplies now or in the future, a caretaker for their cat if they themselves became ill or were unable to play with them, and whether the virus could be passed between cat and guardian. They were also asked to indicate their concern about their ability to keep their cat due to COVID changes. The areas of most concern were the ability to play/exercise with their cat or obtain a caretaker for their cat if they contracted COVID (Table 3). 

Kruskal-Wallis nonparametric analysis of variance found that participants between 40–59 years of age, compared to older and younger guardians, reported greater concern about the ability to afford cat food/supplies now and in the future. Participants 40 years of age and older reported higher concern, compared to younger guardians, about acquiring a caretaker for their cat if they contracted COVID, and the ability to keep their cat because of COVID-related changes. 

Participants were asked if they had identified someone who could care for their cat if they, as guardians, became sick (*n* = 931), to which 580 (62.3%) reported yes, and 351 (37.7%) reported no. Older guardians (50 and older) were more likely to have identified a caretaker than younger guardians (X^2^ = 24.21 (4), *p* < 0.001) (Table 4).

When asked if they had agreed to care for someone else’s cat if that guardian were to become ill (*n* = 931), 116 (12.5%) reported yes, 774 (83.1%) reported no because they had not been asked, and 18 (1.9%) reported no, they had been asked, but could not commit to this responsibility. 

### 3.3. Veterinary Care during COVID

Participants were asked if their cat had required veterinary care since the COVID outbreak (*n* = 931) to which 159 (17.1%) reported yes and 772 (82.9%) reported no. Of those who said they required veterinary care, the majority reported taking their cat to a veterinarian (112, 70.9%). Those who reported going to the veterinarian were asked to indicate all the reasons for the veterinary visit(s). The most common reasons were vaccinations, monitoring an illness/disease or wellness exam. The numbers for each type of visit are listed in Table 5.

Participants were asked about the procedures in place when they visited their veterinarian (*n* = 110). The largest percent reported they were met in the parking lot and not allowed inside the hospital (65, 59.1%), followed by allowed in both the reception area and exam room (24, 21.8%), and then allowed in reception area only (8, 7.3%). Most of the ‘other’ responses (13, 11.8%) reported that the protocols changed over time and they had experienced more than one protocol. 

Lastly, participants were asked to describe their most recent veterinary visit experience since the COVID-19 outbreak, during a time where, in most instances, humans were not allowed in the clinics. Several pertinent issues emerged. The most prominent theme reflected participants’ concerns regarding their inability to be present with their cats in the veterinary clinic. Cat guardians felt the need to be with their cat to support and take care of them and experienced stress as a result, even with minor procedures. One participant noted: “*She needed a blood test for her thyroid to ensure we’re giving the right amount of meds, I sat in my car and they took her in and then back out to me. I actually really hated it because I know how stressful the vet is for her, but I couldn’t be there for comfort*.” Another participant noted: “*It was stressful because I wasn’t allowed in which made me feel sad anxious and concerned that my fur baby was going to be under distress because I wasn’t there or handled quickly because of the COVID-19*.” 

Many participants were appreciative of their veterinarian’s communication pertaining to COVID-19 transmission risks and the extra safety precautions their hospitals implemented. As one participant noted: “*My vet … is the best! I had to take my cat there because she had a follow-up appointment to her full teeth extraction surgery. They had precautions in place for COVID- 19 and had me park in front of the clinic. Then they came out to pick up my cat and I just waited for their call to give me a summary and recommendations and then they brought her out with some dental treats. It was seamless*”. Another participant stated: “*When we first took our cat to the vet the virus was just out breaking in our state and as the illness continued over the span of just two weeks we noticed more measures being implemented for patient and worker safety. Our vet and her staff did a great job to still make us feel like we were being taken care of…*” Conversely, some participants noted they felt stressed by the imposed restrictions. For instance: “*I was a bit more anxious as I couldn’t be with my cat and only interacted with vet via phone*.” Another participant stated: “*Outside clinic. On the parking lot. Don’t like it*.” It seems that cat guardians felt more comfort with the veterinary services, despite the separation from their cat, when there was additional consideration for human safety and a high level of interpersonal engagement with the clinic. 

## 4. Discussion

This study was conducted during the initial months of COVID, an unprecedented time of quarantine that caused many a great deal of anxiety and uncertainly about the future. The sudden mandated changes encompassed all areas of daily living including veterinary medicine and the ability to care for companion animals [15,16,17,18], creating concern among pet guardians across the world [28,29,30,31], and cat guardians in particular. Because of the unique aspects of the cat/human relationship [7,9] the impact of COVID on cat guardians to determine if cat related concerns and experiences differ from those of dog guardians was assessed.

The changes accompanying COVID, in which most people began spending more time at home, appears to have positively impacted their relationship with their cat. Over 60% of guardians reported spending more time with their cat and felt that this additional time strengthened their bond. Additionally, even though people reported more time with their cat, most did not report an increase in frustration over any undesirable behaviors. This positive impact on the bond between guardian and companion animal during COVID lockdown times has been noted in previous studies [29,30,32].

When cat guardians were asked about veterinary related concerns, the most common concerns during this time were availability of their veterinarian for both emergency as well as non-emergency care. The concern regarding the availability for emergencies was felt most acutely by cat guardians 18–29 years of age. These results are similar to the sentiments expressed by dog guardians in a recent study whereby younger dog guardians voiced more concern regarding availability of veterinary services than older dog guardians [32]. Perhaps younger guardians are used to nearly all services and products being consistently and reliably available almost instantly (and have this expectation) [33], they were more impacted by these initial uncertain times. 

Veterinary professionals can help mitigate the worries of pet parents by communicating regularly with their clients and reassuring them that they plan to remain open and available. Although many veterinary hospitals have seen an influx of business since the onset of COVID [34,35], and some are struggling to handle the increased caseload, it is more important than ever to prioritize client communication. Communication of this nature does not have to be detailed or time consuming but should utilize different communication modalities. For example, the use of veterinary clinic mobile apps, text messages, Twitter, and Instagram might be best suited for communicating with younger pet guardians, while email, Facebook, printed materials and phone messages might work better with older clients [36]. Sending frequent messages and updates regarding protocols, regardless of modality, might help decrease guardians’ concerns regarding veterinary clinics’ availability. COVID has also created an opportunity to embrace telehealth, the use of technology to remotely deliver health information, education or care [37]. The use of telemedicine to deliver veterinary medical services can benefit patients, clients and the profession [38] and offers innovative solutions in the quest to serve companion animal guardians safely during the pandemic. To that end, The Royal College of Veterinary Surgeons (RSVS) in the United Kingdom, as well as American Animal Hospital Association (AAHA) and The American Veterinary Medicine Association (AVMA) in the United States have published guidelines to help veterinarians incorporate telemedicine safely and legally [39,40]. As COVID continues to impact daily living, as well as when future instances of radical change necessitate societal restrictions occur, the need to communicate availability through all modalities to ease guardians’ concerns will remain important. 

Of nearly equal concern to veterinary service availability was guardians’ fears about being able to afford emergency care in the future, while fewer participants were worried about affording non-emergency care either at the time of the survey nor in the future. Perhaps this fear pertaining to the ability to afford emergency care is why pet insurance companies have noted substantial growth since the onset of COVID. For example, Trupanion, one of the largest pet health insurance companies, reported a 28% increase in revenue the second quarter of 2020 compared to the previous year [35]. 

When queried about their actual veterinary visits during COVID lockdown times, it is interesting to note that although many veterinary hospitals were advised to limit their cases to essential/emergency services, the most common reasons for veterinary visits (listed by the 17% of respondents who indicted they had taken their cat to the veterinarian since COVID), included obtaining vaccinations, wellness exams, and monitoring an illness/disease. This would suggest that many veterinary hospitals (at least in the US) had not limited their services during the first couple months of COVID. Given the wide array of implemented restrictions throughout the US in response to COVID, variance within veterinary hospital protocols is to be expected.

In regard to cat food and supplies, the ability to obtain these items was a noted concern for many guardians, and in fact, even more so than the ability to afford cat food/supplies at the time of the survey as well as in the future. The initial months of COVID were marked by hoarding behaviors of many staples (e.g., toilet paper, baking supplies, cleaning supplies, etc) [41], fueling fears about the ability to acquire needed supplies. The same hoarding behaviors were seen with pet food [42], likely increasing cat guardians’ fears about acquiring the food and supplies needed to keep their cat happy and well cared for. 

While the ability to afford cat food and supplies, both at that time and in the future, was not as great a concern as being able to obtain supplies, it was still a noted concern for nearly 20% of respondents. Guardians ages 40–59 years of age, compared to both older and younger guardians, reported greater concern about the ability to afford cat food/supplies at that time and in the future. A better understanding of the factors that might be influencing the fears for this middle age cohort is warranted. Insuring access to pet food is not only important for pet welfare, it carries implications for human health. As Rauktis et al. found, companion animal food insecurity and scarcity can create human food security problems as people attempt to supplement their pets’ diet with their own food [43]. To address these concerns among all guardians, it is suggested that communities allocate resources to such entities as pet food pantries or other methods in which guardians can obtain cat supplies. The need for these types of resources is not limited to a pandemic response, however, but extends to any form of disaster. Companion animals have been shown to directly impact how people respond and recover to a variety of disasters [44,45,46,47], leading to recent efforts to integrate companion animals into human emergency planning [48]. It has been suggested that companion animals may be useful conduits for communicating with vulnerable populations during times of disasters, helping guide people towards healthy behaviors that facilitate recovery [49]. Similar efforts could be implemented for pandemic related disasters. 

In addition to veterinary related issues and supplies, cat guardians were concerned about the ability to play/exercise with their cat, obtain a caretaker for their cat if they contracted COVID. Participants 40 years of age and older were more likely to report higher concern levels than younger guardians about obtaining a caretaker for their cat if they contracted COVID as well as the ability to keep their cat because of COVID-related changes. Given the nature of COVID, and its disproportionately negative impact on older adults [50], it follows that older guardians would express more concern about what might happen to their cat if they acquired COVID. Although guardians ages 50 and older were more likely to have identified a caretaker than younger guardians, the percentage of these guardians was still less than ideal (62% for guardians 50–59, 71% for guardians 60 and older). The percent of guardians ages 18–49 who had identified a caretaker were approximately 50–57%, comparable to those found for dog guardians [32]. The number of guardians who have not identified a caretaker is of concern. The psychological response to the overwhelming feelings that can accompany COVID, in addition to real and perceived zoonotic risks, may cause some guardians to consider relinquishing or euthanizing their companion animal [51]. As a result, companion animal welfare may suffer [52]. To help mitigate these negative consequences, proactive measures must be taken. It is important that animal organizations, pet food/supply companies and the veterinary field all work together to encourage guardians to identify a pet caretaker. Organizations such as the ASPCA, for example, offer information and resources on their website to help pet guardians create caretaking arrangements, including an analysis of informal vs formal caretaker agreements as well as a disaster preparedness and pet trust information [53]. In addition to online resources, the creation of a local safety community network of cat guardians and short-term housing options might help ameliorate some of cat guardians’ fears.

Limitations of this study are those inherent in online surveys, including the potential bias of those who chose to participate. Participants were primarily female and had to have internet access and feel comfortable completing a survey in English, so care should be taken when generalizing to other populations. Future studies might benefit from including additional questions such as cat age and time of ownership. Additionally, assessing income level might be of value, as guardians with lower incomes might have fewer resources for veterinary care and cat supplies; a problem likely exacerbated by COVID. Other factors such as how long the guardian has had the cat, as well as their cat’s age, would also be helpful.

## 5. Conclusions

Results of this study suggest that cats remain a top priority for cat guardians even during times of tremendous upheaval. During the initial months of COVID when nearly every aspect of daily life changed overnight, cat guardians wanted and needed reassurance that they would be able to continue caring for their cats. As the veterinary profession continues to adapt to COVID restrictions and safety measures, it is hoped that they prioritize client communication to help assuage client fears. 

## Figures and Tables

**Table 1:** Demographics of participants: Country and level of COVID restrictions.

Country		COVID Restrictions	
United States	915 (95.7%)	All non-essential stores/businesses closed and ordered/strongly recommended to stay at home	791 (82.7%)
Bazil	17 (1.8%)	All non-essential stores/businesses closed but no order to stay at home	131 (13.7%)
Canada	4 (0.4%)	Some stores/businesses closed	23 (2.4%)
United Kingdom	8 (0.8%)	No restrictions	7 (0.7%)
Australia	2 (0.2%)	Other	4 (0.4%)
Other	10 (1.0%)		

**Table 2:** Stated concern for veterinary issues.

	Great Concern	Some Concern	No/Minimal Concern	NA
Concern that my vet will not be available if I need them for emergencies	232 (24.8%)	325 (34.8%)	332 (35.5%)	46 (4.9%)
Concern that my vet will not be available if I need them for non-emergencies	162 (17.3%)	335 (35.8%)	388 (41.5%)	50 (5.3%)
Ability to afford emergency veterinary care in the future	161 (17.12%)	258 (27.6%)	467 (49.9%)	49 (5.2%)
Concern about having to leave the house if my cat gets injured or sick	164 (17.5%)	263 (28.1%)	466 (49.8%)	42 (4.5%)
Ability to afford emergency veterinary care now	151 (16.1%)	241 (25.8%)	487 (52.1%)	56 (6.0%)
Ability to afford non-emergency veterinary care now	87 (9.3%)	189 (20.2%)	607 (64.9%)	52 (5.6%)
Ability to afford non-emergency veterinary care in the future	90 (9.6%)	199 (21.3%)	591 (63.2%)	55 (5.9%)

**Table 3:** Stated concern for non-veterinary cat issues and impact of age.

	Great Concern	Some Concern	No/Minimal Concern	NA/Not an Issue	H (df) *p* Value
A caretaker for your cat if you contracted COVID-19 ^1^	275 (29.4%)	262 (28.0%)	349 (37.3%)	49 (5.2%)	13.77 (4), *p* = 0.008
Ability to play with your cat if you get COVID-19	241 (25.8%)	290 (31.0%)	374 (40.0%)	30 (3.2%)	7.39 (4), *p* = 0.117
Ability to keep your cat because of changes due to COVID-19 ^1^	174 (18.6%)	138 (14.8%)	559 (59.8%)	64 (6.8%)	14.31 (4), *p* = 0.006
Ability to obtain (not due to financial reasons) cat food/supplies	113 (12.1%)	310 (33.2%)	470 (50.3%)	42 (4.5%)	6.62 (4), *p* = 0.158
Concern that you could give your cat COVID-19	144 (15.4%)	215 (23.0%)	535 (57.2%)	41 (4.4%)	8.34 (4), *p* = 0.080
Ability to afford cat food/supplies in the future ^1^	54 (5.8%)	150 (16.0%)	676 (72.3%)	55 (5.9%)	14.21 (4), *p* = 0.007
Ability to afford cat food/supplies now ^1^	40 (4.3%)	128 (13.7%)	706 (75.5%)	61 (6.5%)	13.66 (4), *p* = 0.008
Concern that your cat could give you COVID-19	16 (1.7%)	58 (6.2%)	805 (86.1%)	56 (6.0%)	5.60 (4), *p* = 0.231

^1^ Statistically ignisficant age difference.

**Table 4:** Age as a factor in identified caretaker of cat (*n* = 913).

Age	Identified Caretaker
Under 30	38 (56.7%)
30–39	53 (50.5%)
40–49	74 (53.2%)
50–59	153 (61.9%)
60 and older	253 (71.3%)

**Table 5:** Reasons for veterinary visits since the COVID outbreak.

Reasons	
Monitoring an illness/disease	30 (26.7%)
Wellness exam	25 (22.3%)
Vaccinations	22 (19.6%)
Surgery	14 (12.5%)
Serious illness (e.g., cancer)	11 (9.8%)
Skin/allergy	11 (9.8%)
Dental	10 (8.9%)
Vomiting/diarrhea	6 (5.4%)
Urinary Tract Infection (UTI)	5 (4.5%)
Ear/eye	4 (3.6%)
Subcutaneous fluids	3 (2.7%)
Euthanasia	2 (1.8%)
Accident	1 (0.9%)
Other	9 (8.0%)

## Data Availability

Data available by contacting the corresponding author.

## Supplementary Materials

The following are available online at https://www.mdpi.com/2076-2615/11/3/603/s1, File S1: COVID-19 and cats.
