# Supplementary material for: The Impact of COVID on Cat Guardians: Veterinary Issues
Source: Animals (Basel). 2021 Feb 25;11(3):603. doi: 10.3390/ani11030603 (PMC7996145; doi:10.3390/ani11030603)
Supplement: Supplementary file 1 [file animals-11-00603-s001.pdf]

COVID-19 and cats

Cat owners: COVID-19 is impacting all areas of our lives, including our relationships with our companion animals. We are researchers from Colorado State University, Washington State University, University of San Francisco, and Palo Alto University and we are interested in learning how COVID-19 has impacted your relationship with your cat.
We are specifically looking for people who are over the age of 18 and are currently the primary caretaker of at least one cat. If you meet these qualifications, please consider taking the following short (~10 minute) anonymous survey.

What is involved? You will be asked to complete a series of questions as honestly as possible and there are no right or wrong answers. The questionnaire should take no more than 10 minutes to complete. Participation is entirely voluntary. You may quit at any time.

Are there any benefits or risks in my taking part? There are no direct risks or benefits to completing the survey. The survey is voluntary and anonymous and you may stop the survey at any time by closing the window. Data from the survey will be used only for research and will hopefully be published in a journal.

Will my participation be confidential? Yes, all participation will be confidential. The data will be anonymous and will contain no information that could lead to the identity of individuals. Anonymous data will be kept on a password protected computer.

What happens if I change my mind? If you feel you do not wish to continue with the questionnaire, you can close the browser window.

Where can I get more information? If you have questions about this research please contact Dr. Lori Kogan (Lori.Kogan@ColoState.EDU). Any questions about participant rights related to this survey can be directed to CSU IRB (ricro_irb@mail.colostate.edu) or 970 491-1655. This study has been reviewed and approved by the Research Integrity & Compliance Review Office at Colorado State University.

Consent -- I have read and understood the information given above. In consenting, I agree to take part in this research project and agree for my data to be used for the purpose of this study. I understand that my participation is voluntary and I may withdraw at any time.

Note: This survey asks questions related to COVID-19. If this topic causes any personal distress, you can stop the survey at any time by closing your browser. If you would like more information about COVID-19, please visit the CDC website: https://www.cdc.gov/

Nearly every city is experiencing growing restrictions on travel, including those that have orders to stay at home except for food and essential services. As of today, what level of restrictions are currently in place in your city:

- ◯ No current restrictions
- ◯ Some stores/businesses and restaurants closed
- ◯ All non-essential stores/businesses closed but no orders to stay inside/stay at home
- ◯ All non-essential stores/businesses closed and order to stay inside/stay at home
- ◯ Other ____

Please tell us a bit about your household in the following questions.

In what country do you currently live?

- ◯ United States
- ◯ Canada
- ◯ UK
- ◯ Australia
- ◯ Other (please specify) ____

How many adults regularly live in your home (including yourself)?

▼ 1 ... >5

How many children (under 18 years of age) live in your home?

▼ 0 ... >5

How many cats live in your home?

▼ 1 ... >5

What is your age?

____

Please indicate your gender:

- ◯ Male
- ◯ Female
- ◯ Non-binary
- ◯ NA

What is your current work status:

- ◯ I have always worked from home and am working from home now
- ◯ I was working outside the home and am now working from home
- ◯ I was working outside the home and am still working outside the home
- ◯ I was working outside the home and have been laid off
- ◯ I have always worked from home and have been laid off
- ◯ Other: ______________________

Please think back to one month ago, right before the COVID 19 outbreak – how would you describe the availability of your social support system?

- ◯ Minimal available social support
- ◯ Some available social support
- ◯ A great deal of available social support

Please indicate the availability of your social support system at this time:

- ◯ Minimal available current social support
- ◯ Some available current social support
- ◯ A great deal of available current social support

Have you been diagnosed with COVID-19?

- ◯ Yes
- ◯ No

Do you suspect you have had (or currently have) COVID-19, but have not been tested?

- ◯ Yes (please explain why): ____
- ◯ No

Have you been ordered to quarantine?

- ◯ Yes
- ◯ No

Have you placed yourself in self-quarantine?

- ◯ Yes
- ◯ No

For the following questions, if you have more than one cat, please answer the questions about the cat whose name starts with a letter closest to the beginning of the alphabet (for example, choose to answer about Fluffy rather than Sylvester since "F" comes before "S" in the alphabet).

Please think back to one month ago, right before the COVID 19 outbreak - how would you have rated your bond with your cat on a scale from 1 (not at all bonded) to 10 (extremely bonded)?

▼ 1 (not bonded at all) ... 10 (extremely bonded)

Now, thinking about today, how would you rate your bond with your cat on a scale from 1 (not at all bonded) to 10 (extremely bonded)?

▼ 1 (not bonded at all) ... 10 (extremely bonded)

Most cats have at least some behaviors that can be frustrating at times. Please think back to one month ago, right before the COVID 19 outbreak - how frustrated did you feel about your cat's undesirable behavior(s) on a scale from 1 (minimal/no frustration) to 10 (extremely frustrated)?

▼ 1 (minimal/no frustration) ... NA (no undesirable behaviors)

Now, thinking about today, how frustrated do you feel about your cat's undesirable behavior(s) on a scale from 1 (minimal/no frustration) to 10 (extremely frustrated)?

▼ 1 (minimal/no frustration) ... NA (no undesirable behaviors)

Please indicate the impact (if any) of COVID-19 on the time you spend with your cat (actively engaging or just being in the house together):

- ◯ Decreased amount of time
- ◯ Same amount of time
- ◯ Increased amount of time

Do you feel the increased amount of time you are spending with your cat is strengthening your relationship or creating strain in the relationship?

- ◯ Strengthening (please explain) ____________________
- ◯ Straining (please explain) ____________________
- ◯ Both strengthening and straining (please explain) ____________________
- ◯ No change

Please indicate the impact of COVID-19 on the time you spend actively engaging with your cat in the house or yard (playing, petting, sitting/laying together):

- ◯ Decreased amount of time
- ◯ Same amount of time
- ◯ Increased amount of time

Please indicate how your cat has impacted EACH of the following feelings you might be having related to COVID-19.

	Greatly decreased	Decreased	No change/impact	Increased	Greatly increased	NA/Is not an issue
Feelings of anxiety	◯	◯	◯	◯	◯	◯
Feelings of depression	◯	◯	◯	◯	◯	◯
Feeling overwhelmed	◯	◯	◯	◯	◯	◯
Feeling isolated	◯	◯	◯	◯	◯	◯
Feeling lonely	◯	◯	◯	◯	◯	◯
Ability to maintain a regular schedule	◯	◯	◯	◯	◯	◯
Ability to cope with uncertainty	◯	◯	◯	◯	◯	◯
Give purpose /meaning to your life	◯	◯	◯	◯	◯	◯
Compassion towards myself	◯	◯	◯	◯	◯	◯

Please indicate your concern level with EACH of the following veterinary-related issues as they relate to COVID-19.

	No concern	Minimal concern	Some concern	Great concern	NA/Is not an issue
Ability to afford emergency veterinary care now	○	○	○	○	○
Ability to afford emergency veterinary care in the future	○	○	○	○	○
Ability to afford non-emergency veterinary care now	○	○	○	○	○
Ability to afford non-emergency veterinary care in the future	○	○	○	○	○
Concern that my vet will not be open/available if I need them for emergencies	○	○	○	○	○
Concern that my vet will not be open/available if I need them for non-emergencies	○	○	○	○	○
Concern about having to leave the house if my cat gets injured or sick	○	○	○	○	○

Please indicate your concern level with EACH of the following cat-related issues as they relate to COVID-19.

	No concern	Minimal concern	Some concern	Great concern	NA/Is not an issue
Ability to afford cat food/supplies now	○	○	○	○	○
Ability to afford cat food/supplies in the future	○	○	○	○	○
Ability to obtain (not due to financial reasons) cat food/supplies	○	○	○	○	○
A caretaker for your cat if you contracted COVID-19	○	○	○	○	○
Concern that you could give your cat COVID-19	○	○	○	○	○
Concern that your cat could give you COVID-19	○	○	○	○	○
Ability to play with your cat if you get COVID-19	○	○	○	○	○
Ability to keep your cat because of changes due to COVID-19	○	○	○	○	○

There are many stressors that can come with COVID-19. Do you feel that having a cat adds, reduces, or has no impact on your stress level? Please explain:

____

Have you made a plan or identified someone who could care for your cat if you got sick?

- ◯ Yes
- ◯ No

Have you agreed to care for someone else's cat if they got sick?

- ◯ Yes
- ◯ No, I have not been asked
- ◯ No, I have been asked, but I feel unable to commit to this
- ◯ Other: ____

Has your cat needed veterinary care since the COVID 19 outbreak?

- ◯ Yes
- ◯ No

Have you taken your cat to the veterinarian since the COVID 19 outbreak?

- ◯ Yes
- ◯ No

How many times have you taken your cat to the veterinarian since the COVID 19 outbreak?

▼ 1 ... >5

Please indicate for what reason(s) you have taken your cat to the veterinarian since the COVID 19 outbreak? (select all that apply)

- ☐ Vaccinations
- ☐ Wellness exam
- ☐ Dental
- ☐ Skin/allergy
- ☐ Surgery
- ☐ Accident
- ☐ Vomiting/diarrhea
- ☐ Serious illness (e.g., cancer)
- ☐ Monitoring an illness/disease/recheck
- ☐ Euthanasia
- ☐ Other ____

Were you allowed in the veterinary clinic for this appointment or did you interact in the parking lot/outside only?

- ◯ Allowed in clinic reception area and exam room
- ◯ Allowed in clinic reception area only
- ◯ Was met in the parking lot/outside, not allowed in veterinary clinic
- ◯ Other: ____

Please describe your most recent veterinary visit experience since the COVID 19 outbreak:

____

Please indicate your concern level, because of COVID-19 changes, about the ability to provide for **your own** basic needs (home, food, medical care, etc)?

- ◯ Not at all concerned
- ◯ Minimal concern
- ◯ Some concern
- ◯ A great deal of concern

Please indicate your concern level, because of COVID-19 changes, about the ability to provide for **your cat's** basic needs (home, food, veterinary care, etc)?

- ◯ Not at all concerned
- ◯ Minimal concern
- ◯ Some concern
- ◯ A great deal of concern

Please share any additional thoughts about how COVID-19 as impacted your relationship with your cat:

____

Have you adopted a cat since the COVID 19 outbreak?

- ○ Yes
- ○ No

Please explain why you decided to adopt a cat since the COVID 19 outbreak:

____

When did you make the decision to adopt a cat?

- ○ Before the COVID-19 outbreak
- ○ Since the time of the COVID-19 outbreak

Do you plan to adopt a cat within the next month?

- ○ Yes
- ○ Maybe
- ○ No

Please explain why you plan to adopt a cat in the next month:

____

Have you fostered a cat since the COVID 19 outbreak?

- ○ Yes
- ○ No

Why have you chosen to foster a cat since the COVID 19 outbreak?

Do you plan to foster a cat within the next month?

- ○ Yes
- ○ Maybe
- ○ No

Why do you plan to foster a cat in the next month?

When did you make the decision to foster a cat?

- ○ Before the COVID-19 outbreak
- ○ Since the time of the COVID-19 outbreak

Do you plan to continue fostering if you return to on-site work?

- ◯ I do not plan to return to on-site work
- ◯ Yes, I plan to continue fostering if I return to on-site work
- ◯ No, I do not plan to continue fostering if I return to on-site work
- ◯ Other (please explain) ____

Before the COVID-19 outbreak, did you actively volunteer for an animal shelter/rescue (outside of fostering animals)?

- ◯ Yes
- ◯ No

After the COVID-19 outbreak, do you plan to volunteer for an animal shelter/rescue (outside of fostering animals)?

- ◯ Yes
- ◯ No

Now that you have completed the survey, please tell us anything else you would like to about how COVID-19 has impacted your relationship with your cat or your feelings about living with a cat.

____

Thank you!
